# Deep-Learning-Based Hemoglobin Concentration Prediction and Anemia Screening Using Ultra-Wide Field Fundus Images

**DOI:** 10.3389/fcell.2022.888268

**Published:** 2022-05-19

**Authors:** Xinyu Zhao, Lihui Meng, Hao Su, Bin Lv, Chuanfeng Lv, Guotong Xie, Youxin Chen

**Affiliations:** ^1^ Department of Ophthalmology, Peking Union Medical College Hospital, Chinese Academy of Medical Sciences, Beijing, China; ^2^ Key Lab of Ocular Fundus Diseases, Chinese Academy of Medical Sciences, Beijing, China; ^3^ Ping An Healthcare Technology Company Limited, Shenzhen, China; ^4^ Ping An Health Cloud Company Limited, Shenzhen, China; ^5^ Ping An International Smart City Technology Company Limited, Shenzhen, China

**Keywords:** anaemia, hemoglobin, ocular fundus, deep learning, ultra-wide-field fundus images

## Abstract

**Background:** Anemia is the most common hematological disorder. The purpose of this study was to establish and validate a deep-learning model to predict Hgb concentrations and screen anemia using ultra-wide-field (UWF) fundus images.

**Methods:** The study was conducted at Peking Union Medical College Hospital. Optos color images taken between January 2017 and June 2021 were screened for building the dataset. ASModel_UWF using UWF images was developed. Mean absolute error (MAE) and area under the receiver operating characteristics curve (AUC) were used to evaluate its performance. Saliency maps were generated to make the visual explanation of the model.

**Results:** ASModel_UWF acquired the MAE of the prediction task of 0.83 g/dl (95%CI: 0.81–0.85 g/dl) and the AUC of the screening task of 0.93 (95%CI: 0.92–0.95). Compared with other screening approaches, it achieved the best performance of AUC and sensitivity when the test dataset size was larger than 1000. The model tended to focus on the area around the optic disc, retinal vessels, and some regions located at the peripheral area of the retina, which were undetected by non-UWF imaging.

**Conclusion:** The deep-learning model ASModel_UWF could both predict Hgb concentration and screen anemia in a non-invasive and accurate way with high efficiency.

## Introduction

Anemia is the most common hematological disorder that affects an estimated two billion people globally (Kassebaum and Collaborators, 2016). The causes involve various diseases such as malnutrition, malignancy, chronic kidney disease, and gastrointestinal bleeding, with iron deficiency being the most common one. Anemia impairs the health, well-being, and cognitive function of people (GBD DALYs and HALE Collaborators, 2018), leading to decreased physical work, fatigue, and increased susceptibility to infection, low birth weight, and jeopardizing child’s physical and mental development (Milman, 2011). Severe anemia might cause various cardiac, renal, and cerebral diseases or even malignant tumors. Fortunately, anemia is usually correctable. Therefore, timely screening and early intervention are crucial for a favorable prognosis.

Current methods to screen anemia or measure blood HGB levels include two categories: invasive and non-invasive. The gold standard of all methods is decreased hemoglobin (Hgb) concentration in the blood, which is relatively simple, effective, and accepted worldwide (Tyburski et al., 2014). However, getting the blood sample is invasive and painful and requires a trained phlebotomist, a clinical hematology analyzer with trained laboratory technicians, biochemical reagents, and infrastructure. These make it inconvenient when screening anemia in quite a large population or in rural areas. Other non-invasive and rapid techniques include deep-learning-based approaches from electrocardiograms (ECGs), analyzing color and metadata of fingernail bed smartphone apps and extracting photoplethysmogram characteristics of fingertip video (Mannino et al., 2018; Kwon et al., 2020; Golap et al., 2021). Unfortunately, none of them is expanded due to their limitations, such as not being suitable for patients with right ventricular diseases using conventional ECGs, relatively small sample size, and selection bias in the other two reports.

Eyes can reflect the condition and function of different organs, such as cardiac, cerebral, and renal disorders. Anemia also has tight connections with ocular manifestations. Several studies have explored to screen anemia using conjunctival features based on the phenomenon of conjunctival pallor (Collings et al., 2016; Batsis et al., 2019; Kasiviswanathan et al., 2020). In addition, anemia could cause various fundus changes, such as retinal hemorrhages, soft exudates, venous tortuosity, ischemic retinopathy, and papilledema (Aisen et al., 1983). For moderate anemia, the disturbance of retinochoroidal circulation and structural stability had also been noticed (Cikmazkara and Ugurlu, 2016; Simsek et al., 2016). Therefore, we assumed that a comprehensive evaluation of ocular fundus features could help in screening the anemia and prediction of the Hgb value. Mitani et al (2020) and Tham et al (2020) trained a deep-learning model to realize the automated anemia screening based on retinal fundus images. The results were encouraging, and they revealed that blood vessels near the optic disc are the areas of the highest interest in the deep-learning model. Wei et al (2021) proposed a lightweight network to screen anemia from retinal vessel optical coherence tomography (OCT) images. However, the retinal fundus photography is just capable of acquiring 30°–60° views of the posterior retina to routinely evaluate macula and optic disc. The OCT is not the primary choice to evaluate the retinal blood vessels. Therefore, a more comprehensive, sensitive, feasible, and non-invasive strategy to screen and predict anemia by fundus images is needed. Ultra-wide-field (UWF) scanning laser ophthalmoscopy enables capturing the ocular fundus up to 200° in a single exposure (Yoo et al., 2020), offering us an opportunity to solve the aforementioned problems. Previous studies have successfully developed deep-learning-based techniques to identify the lattice degeneration, retinal detachment, or other notable peripheral retinal lesions based on UWF fundus images (Ohsugi et al., 2017; Li et al., 2020; Zhang et al., 2021). While still, no study focuses on deep-learning-based anemia prediction by UWF fundus images.

The purpose of this study was to develop and validate a deep-learning algorithm for anemia screening by UWF fundus images. We first collected a larger number of UWF fundus images with corresponding Hgb values to develop a deep-learning-based model for hemoglobin concentration prediction and anemia screening simultaneously. Then, we cropped the center part of the original UWF fundus images to train a new deep-learning model and compared its performance with the model of original UWF fundus images, to investigate whether the peripheral area of the retina was contributable to the prediction of hemoglobin concentration and anemia. We hope this technique may service as a comprehensive, sensitive, and non-invasive approach to screen and predict anemia, which could be used in a large population screening and fit for rural application.

## Materials and Methods

### Study Design and Participants

The dataset was generated from January 2017 to June 2021 in Peking Union Medical College Hospital (PUMCH). Optos (Daytona, Optos PLC, Dunfermline, United kingdom) color images taken during this period were screened by two retinal specialists (Xinyu Zhao and Lihui Meng) independently. The inclusion criteria were: 1) at least one Optos image; 2) paired with Hgb measurement within 1 week when the Optos images were taken; 3) no transfusion therapy or blood donation during the interval between images taken and Hgb measurement; 4) for one subject who visited the clinics for multiple times, only those that had an interval beyond 6 months between two visits. The exclusion criteria were: 1) severe artifact; 2) blurred images due to vitreous hemorrhage, astrocytosis, or intense cataracts; 3) retinal vessels, optic disc, and other fundus structures could not be shown clearly; 4) patients who underwent previous vitreoretinal surgery or retinal photocoagulation; 5) brightness that was not too dark or too light. The Hgb was obtained by the hemoglobincyanide (HiCN) method. This study was approved by the Ethics Committee of PUMCH, Chinese Academy of Medical Sciences. The procedure was adhered to the tenets of the Declaration of Helsinki, and the need for patient consent was waived by the Institutional Review Boards at PUMCH.

According to guidelines from the WHO (Davidson et al., 2011), the definitions of anemia and classifications of severity were as follows: the cut-off value of anemia was 12 g/dl for women and 13 g/dl for men.

### Development of the Deep-Learning-Based Anemia Screening Model

**FIGURE 1:**
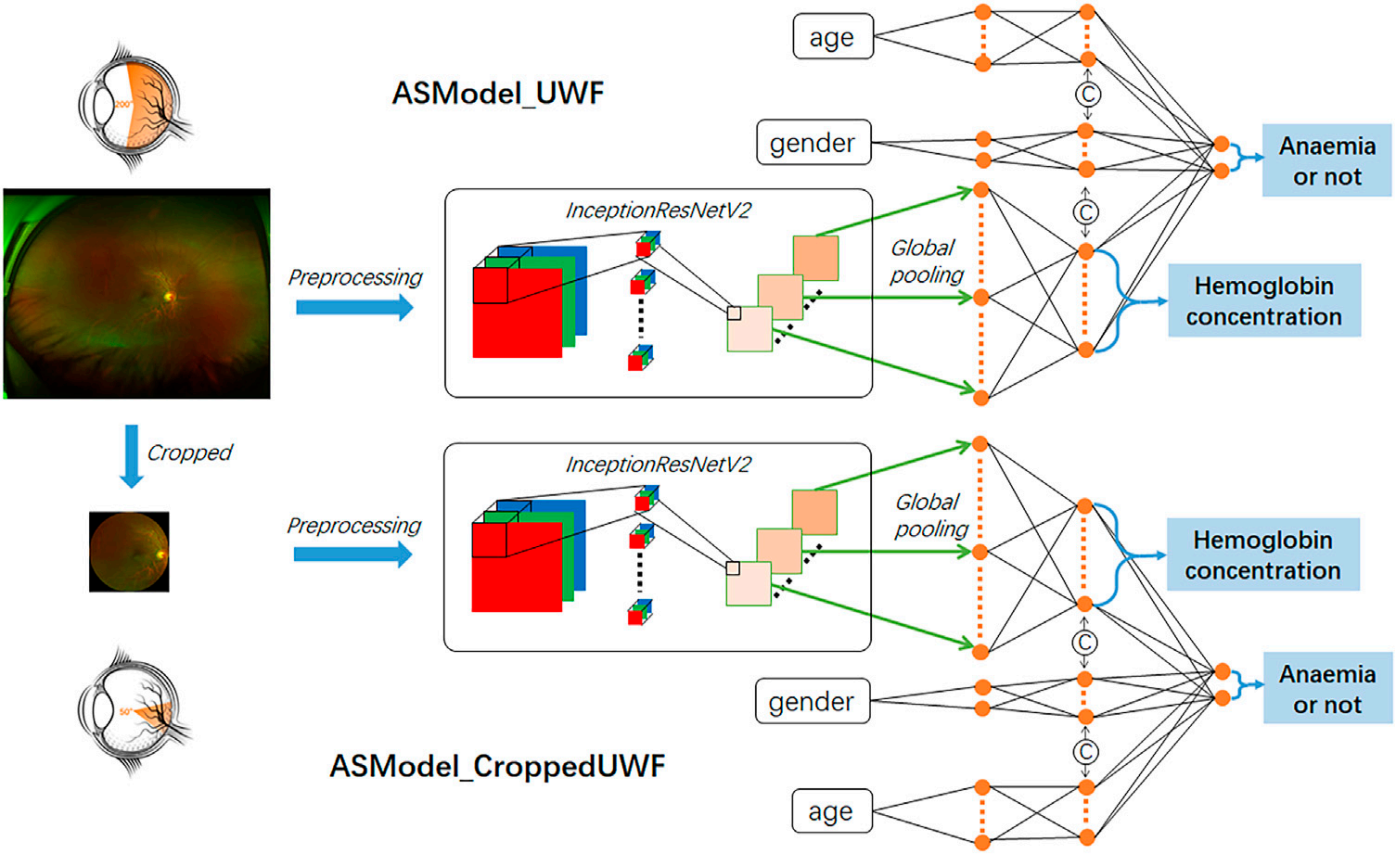
Illustration of our constructed network architecture for hemoglobin concentration prediction and anemia screening based on UWF fundus images. ASModel_UWF is the model trained by original UWF fundus images, while ASModel_CroppedUWF is the model trained by the cropped UWF images.

The constructed anemia screening model was a multitask cascaded convolutional neural network, while the first task is hemoglobin concentration prediction based on UWF fundus images and the second task is to determine the presence or absence of anemia by integrating the demographic information (age and gender). We selected InceptionResNetV2 as the main architecture for the first task which combined the advantages of the inception network and residual network to improve the network performance (Szegedy et al., 2017). UWF fundus images were first transformed into feature maps by InceptionResNetV2 and further converted into feature vectors by the global pooling layer. The hemoglobin concentration was then predicted by the fully connected layers with 17 nodes, while the prediction range was from 4 g/dl to 20 g/dl. For the second cascaded task, age and gender information would be concatenated with the fully connected layers to achieve the final anemia screening. The categorical input of gender was represented as two-class one-hot vectors, and the continuous input of age was transformed into the seven-digit binary numbers (from 0 to 128 years old). The network structure of the constructed model is presented in Figure 1.

Weights pre-trained for the image classification task with ImageNet dataset (Krizhevsky et al., 2012) were used to initialize the InceptionResnetV2 network, and other fully connected layers for inputs and outputs were randomly initialized. Before training the constructed model, all UWF fundus images were preprocessed with the contrast limited adaptive histogram equalization (CLAHE) method (Pizer et al., 1987) for image quality improvement and then resized into 512 × 512 with bicubic interpolation. The routine data augmentation methods (e.g., horizontal and vertical flipping and rotation up to 30°) were applied to increase the training dataset, thus reducing the possibility of overfitting. We designed a loss function consisting of the mean squared error (MSE) and cross-entropy error (CE) for training the model. MSE was used for the task of hemoglobin concentration prediction, and CE was used for the anemia screening task. The optimizer of the algorithm was Adam with a learning rate of 1e-3 and L2 weight decay of 5e-4. Early stopping was also used to prevent overfitting based on the mean average error on the validation dataset. We implemented the deep-learning network with the PyTorch framework in Python 3.6.

### Performance Evaluation of the Developed Model

As our deep-learning model has two outputs, one is a continuous prediction of hemoglobin concentration and another is a categorical prediction of whether it is anemia or not, we therefore need to evaluate the model performance in different ways. For the continuous prediction of hemoglobin concentration, we used the mean absolute error (MAE) with 95% limit of agreement. For the categorical prediction of the possibility of anemia, we used the area under the receiver operating characteristics curve (AUC) and sensitivity at various levels of specificity to measure the performance of the algorithm.

UWF fundus images can capture the peripheral retina rather than just the posterior pole and optic nerve area. To investigate whether the peripheral retina contributed to anemia screening, we cropped the central area from original UWF fundus images to generate the images having a similar view field of traditional fundus images and trained another anemia screening model (named ASModel_CroppedUWF) with these cropped images as the inputs of the network. The mask for cropping was a circle with the midpoint as the center and the length of 25% image horizontal width as the diameter, and the same image preprocessing steps were applied during model training and validation. We then compared the model performance of ASModel_CroppedUWF with the model trained by original UWF fundus images (ASModel_UWF).

In addition, we also compared our developed models with other invasive or non-invasive anemia screening methods in multiple aspects. First, we compared the selected data types and the corresponding techniques and the test/total experimental dataset since the dataset size is closely related to the stability of actual performance. Second, we applied MAE to reflect the performance of hemoglobin concentration prediction and used AUC, sensitivity, and specificity to evaluate the anemia screening results for different methods.

### Model Interpolation

To highlight the regions in UWF fundus images on which our deep-learning models focused the most while predicting hemoglobin concentration or detecting anemia, we generated the saliency maps with the visual explanation tools of GradCAM (Selvaraju et al., 2017) and Guided-Backpropagation (Springenberg et al., 2014). GradCAM was applied to the final convolutional layer so that it could generate heatmaps with the same resolution algorithm’s output. The more effective that the region makes on predictions, the redder it will be in heatmaps (Selvaraju et al., 2017). Guided-Backpropagation could show the region that positively contributed to the prediction by tracking gradients. This method ignores the negative gradients in each layer of the algorithm and traces back to the specific location on the input images following the positive gradients (Springenberg et al., 2014). Both methods can highlight the contributions of parts of the networks to regions in the image and present them by covering the colored heatmaps on original images to give a visual indicator of the important region in the image.

## Results

**FIGURE 2:**
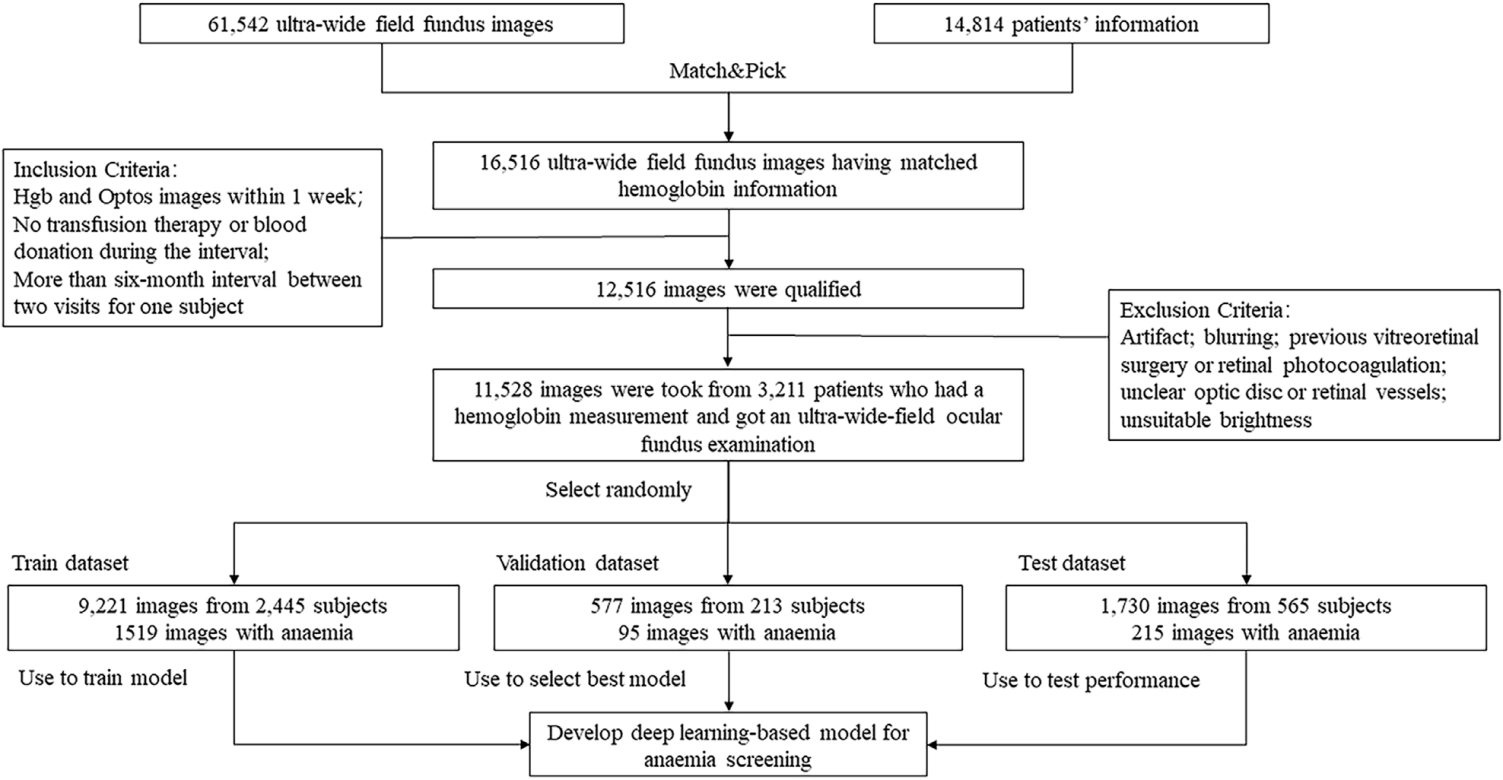
Study profile of dataset selection.

**FIGURE 3:**
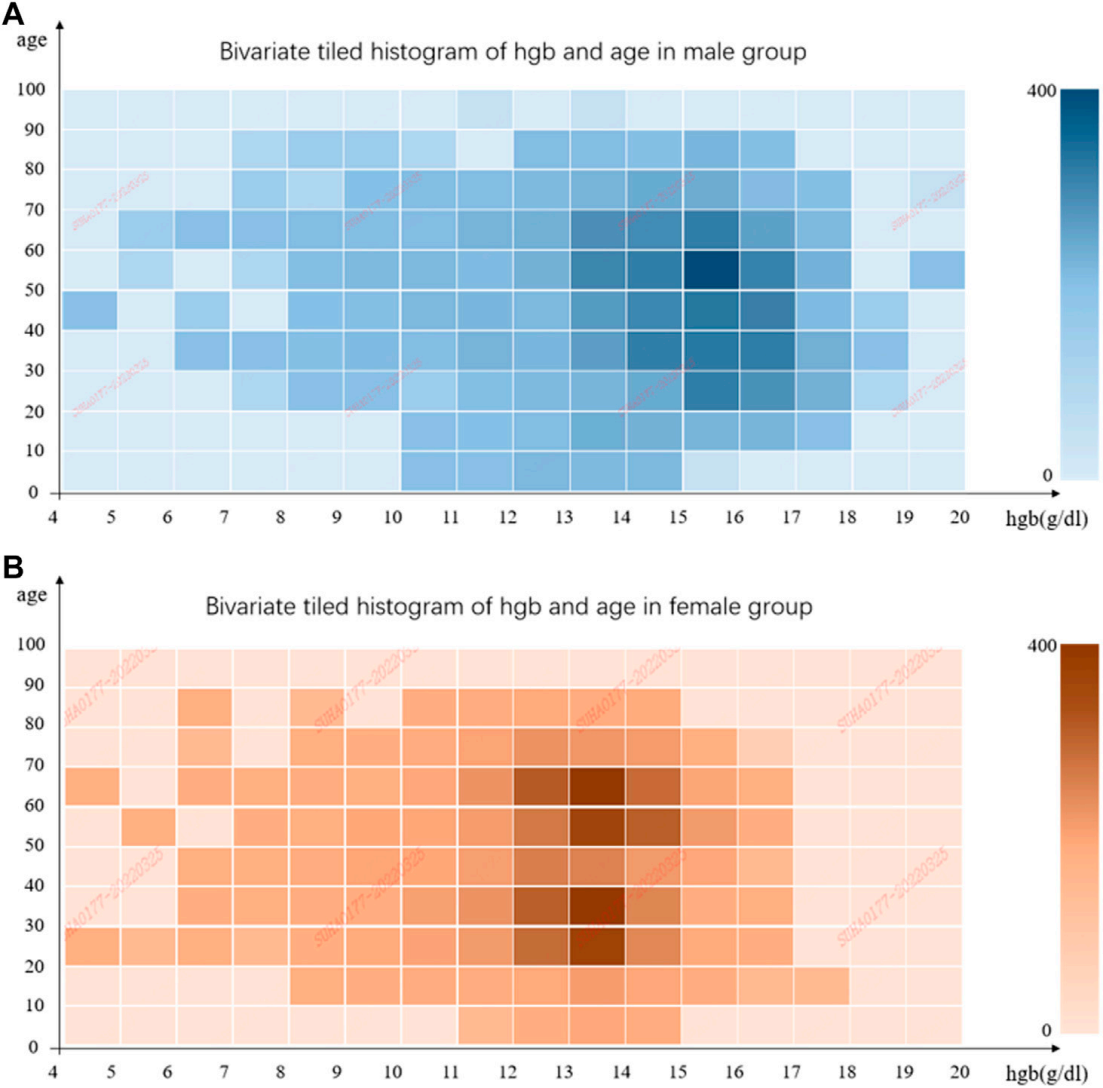
Bivariate tiled histograms of hemoglobin concentration and age in male and female groups. **(A)** is for male and **(B)** is for female. The abscissa represents hemoglobin concentration, and the ordinate represents age. Each bin covers 1 g/dl at the horizontal coordinate, and 10 years old at the vertical coordinate, and the color of bins indicates the number of elements in them.

In total, we collected 61,542 UWF fundus images from 14,814 patients who were admitted to PUMCH from January 2017 to July 2021. About 4,512 patients did hemoglobin measurements, paired with 16,500 images. To make sure UWF fundus images correspond well with Hgb correctly and timely, the images which were not paired with corresponding hemoglobin measurement in 2 weeks were excluded. Then, blurred or low-quality images were discarded, as mentioned previously. Finally, our dataset included 11,528 images from 3,211 patients. The flowchart is shown in Figure 2. We also illustrated the distributions of hemoglobin concentration and age for the male and female groups in Figure 3. According to the bivariate tiled histograms, we could find that the male group had higher hemoglobin concentration than the female group in our dataset.

We then divided all UWF images into the train set (9,221 images), validation set (577 images), and test set (1,730 images). We used data augmentation, including horizontal image flip and random rotation within 5 to −5°, to increase the train set to 27,663 images. The median ages of subjects (with interquartile range) are 44 (33–61), 43 (26–61), and 43 (27–57) years for three datasets. Additional demographical information is shown in Supplementary Table S1. The images from the same subject in a single visit were put in the same dataset, to make sure these images of the same subject would not be used in both training and testing.

**FIGURE 4:**
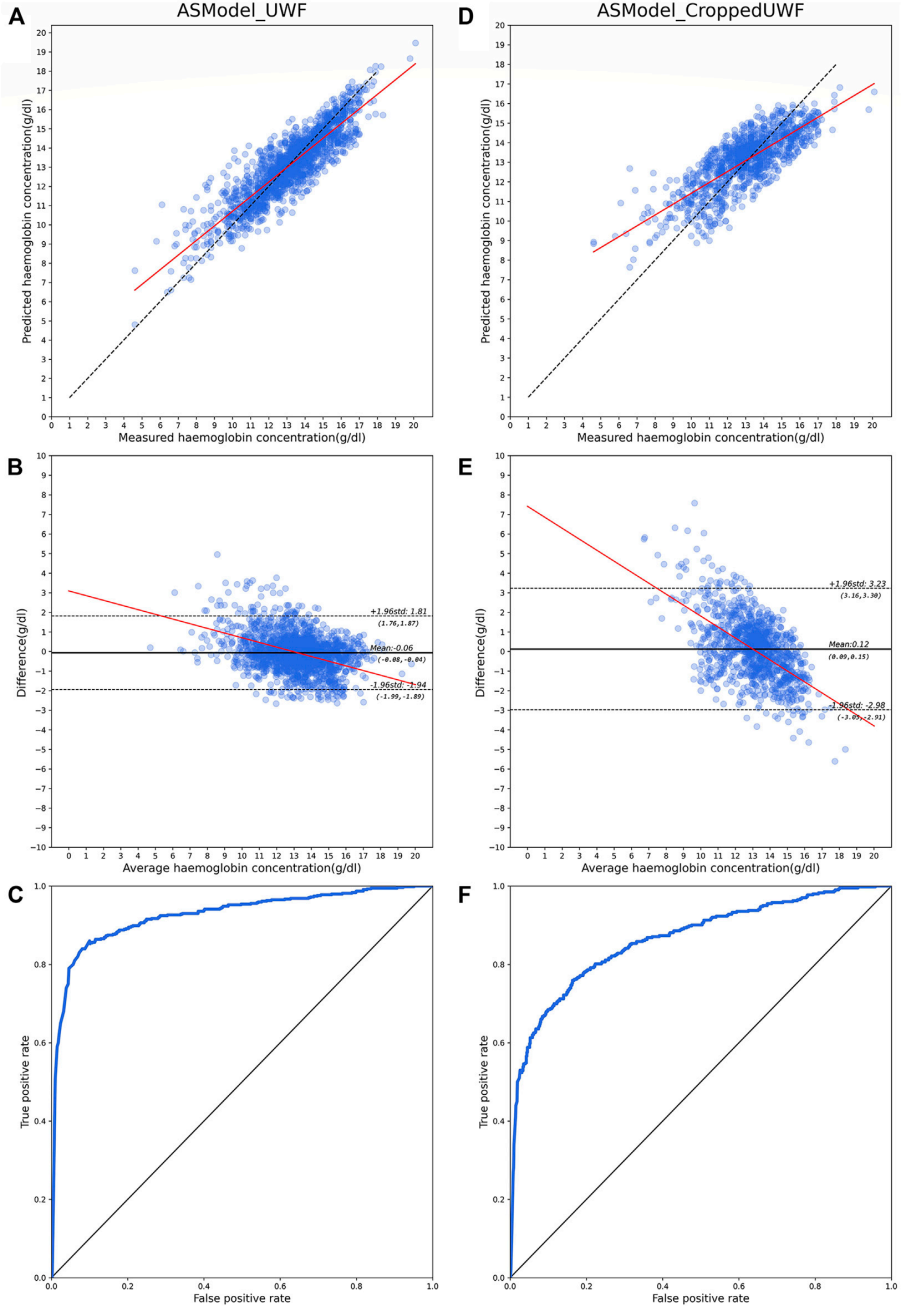
Model performance for hemoglobin concentration prediction and anemia screening with UWF fundus images and cropped images. **(A–C)** are the results for ASModel_UWF, while **(D–F)** are the results for ASModel_CroppedUWF. **(A,D)** Scatter diagrams for hemoglobin concentration prediction. Each dot represents the predicted value against the measured value, and the black broken line represents the ideal model, and the red line represents the linear fit of results. **(B,E)** Bland–Altman plots for predicted and measured hemoglobin concentration. Each dot represents the difference predicted value and measured value against the measured value. The black line represents the mean value of the difference, the black broken lines represent 95% limits of agreement, and the red line represents the line fit. **(C,F)** ROC curves for anemia screening.

The performance of hemoglobin concentration prediction and anemia screening is shown in Figure 4. Figures 4A and B are the predicted hemoglobin concentration results in the test dataset with ASModel_UWF, while Figures 4D and E are the results for ASModel_CroppedUWF. Figures 4A,D were the scatter diagrams, and Figures 4B,E were the Bland–Altman plots for the predicted and measured hemoglobin concentration. The linear fit’s slope of hemoglobin prediction was 0.76 (95% CI: 0.75–0.77) for ASModel_UWF (Figure 4A) and 0.55 (95% CI: 0.53–0.57) for ASModel_CroppedUWF (Figure 4D). Also, the MAE of prediction task was 0.83 g/dl (95% CI: 0.81–0.85 g/dl) for ASModel_UWF and 1.21 g/dl (95% CI: 1.16–1.26 g/dl) for ASModel_CroppedUWF. From the Bland–Altman plots (Figures 4B and E), we observed that the negative slope in the linear fit was −0.23 (95% CI: 0.25 to −0.21) for ASModel_UWF and was −0.56 (95% CI: 0.59 to −0.52) for ASModel_CroppedUWF, which indicated there was a proportional bias for both models. Figure 4C showed the receiver operating characteristics curve of anemia screening for ASModel_UWF and ASModel_CroppedUWF. The algorithm trained by UWF fundus images had an AUC of 0.93 (95% CI: 0.92–0.95), and the AUC for the cropped UWF fundus images was 0.86 (95% CI: 0.85–0.89).

The comparison of different anemia screening methods is shown in Table 1. We compared our developed models with invasive measurement (Gehring et al., 2002) and other non-invasive approaches. The non-invasive approaches included fingernail photo (Mannino et al., 2018), conjunctiva photo (Bauskar et al., 2019; Kasiviswanathan et al., 2020), OCT image (Wei et al., 2021), ECG signal (Kwon et al., 2020), traditional fundus image (Mitani et al., 2020), and our UWF fundus image. The compared dimensions included test/total dataset, MAE, AUC, sensitivity, and specificity. We investigated the sensitivity of our models (ASModel_UWF and ASModel_CroppedUWF) at 80% specificity, in order to facilitate the comparison with previous methods (Kwon et al., 2020; Mitani et al., 2020). The results showed that our ASModel_UWF achieved the best performance of AUC and sensitivity among the methods whose test dataset sizes were over 1000.

**TABLE 1:** Performance comparison for different methods of hemoglobin prediction and anemia screening.

Data type	Method	Test/total dataset	MAE (g/dl)	AUC	Sensitivity*	Specificity
Hematology analyzer	Invasive measurement with Blood analyze	\	0.14	\	\	\
Fingernail photo	Deep-learning	50/337	0.41	0.94	92.00%	76.00%
Conjunctiva photo	Deep-learning	18/99	\	\	100%	88.00%
Conjunctiva photo	Ridge regression	27/135	0.99	\	\	\
OCT	Deep-learning	343/1369	\	0.998	98.40%	95.90%
ECG signal	Deep-learning	12,639/70,074	0.79 (0.78–0.80)	0.92 (0.91–0.94)	89.80%	81.50%
Fundus image	Deep-learning	22,742/114,205	0.63	0.89	79.50%	80.00%
ASModel_CroppedUWF	Deep-learning	1730/11,528	1.21 (1.16–1.26)	0.86 (0.85–0.89)	78.7% (74.3–82.5%)	80.00%
ASModel_UWF	Deep-learning	1730/11,528	0.83 (0.81–0.85)	0.93 (0.92–0.95)	91.2% (88.4–93.2%)	80.00%

*Sensitivities are related to specificities on the right.

**FIGURE 5:**
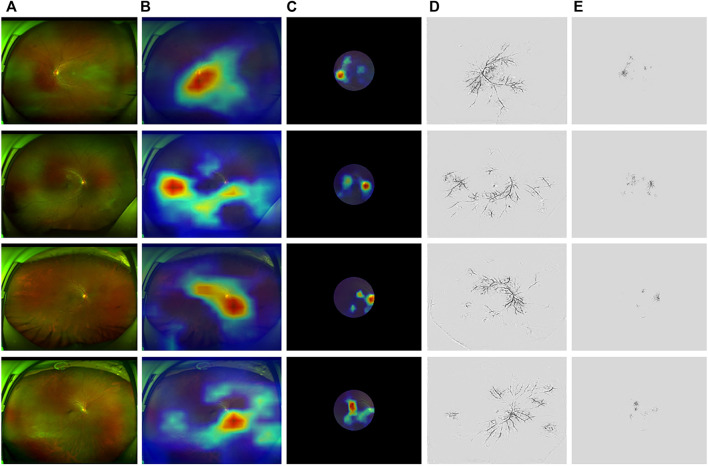
Examples applying GradCAM and Guided-Backpropagation to highlight the region’s model while focusing on predicting. **(A)** Original UWF fundus images. The region in the red circle is the same as cropped images. **(B,C)** Saliency maps of ASModel_UWF and ASModel_CroppedUWF from GradCAM covering the original and cropped UWF images, respectively. **(D,E)** Saliency maps of ASModel_UWF and ASModel_CroppedUWF from Guided-Backpropagation, respectively. The top two rows were normal subjects (64 years old, male, 17 g/dl; 12 years old, male, 14.1 g/dl), while the below two rows were anemia patients (58 years old, male, 8.6 g/dl; 44 years old, female, 10.2 g/dl).

Figure 5 illustrates the results with model interpolation techniques on ASModel_UWF and ASModel_CroppedUWF, respectively. Figures 5B andC were the results visualized by GradCAM, and Figure 5D and E were the results of Guided-Backpropagation. We could observe that both ASModel_UWF and ASModel_CroppedUWF tend to focus on the spatial features around the optic disc and retinal vessels, just like the previous study based on the fundus image (Mitani et al., 2020). However, ASModel_UWF also paid attention to some regions located at the peripheral area of the retina, which could not be detected by non-UWF imaging. The comparison between different models indicated that these peripheral regions contribute to the better performance of our ASModel_UWF.

## Discussion

Our study developed a deep-learning model, ASModel_UWF, using UWF images to predict Hgb concentration and screen anemia. The MAE of the prediction task was 0.83 g/dl (95% CI: 0.81–0.85 g/dl), and the AUC of the screening task was 0.93 (95% CI: 0.92–0.95). Compared with other techniques, ASModel_UWF achieved the best performance of AUC and sensitivity when the test dataset size was larger than 1000.

As shown in Figure 5, fine spatial features around the optic disc were crucial for anemia prediction, which was consistent with the previous study by Mitani et al. (2020). This finding could be supported by some biological phenomena and observations. For example, patients with iron deficiency anemia (IDA) were reported to have significantly thinner peripapillary retinal nerve fiber layer (RNFL) thickness, and the Hgb levels were also significantly correlated with the average RNFL thickness value (Türkyilmaz et al., 2013; Akdogan et al., 2015). Additionally, optic atrophy, disc edema, and epiretinal tissue on the optic disc have been identified in anemia patients (Sethi et al., 2020; Ab Gani et al., 2021; Graf et al., 2021), which suggested that the optic disc was susceptible to be affected by anemia. Moreover, ASModel_UWF paid attention to the peripheral retina. The results suggested that the deep-learning model could achieve higher performance when the peripheral retina of UWF images was included. The saliency maps explained the difference between UWF and normal fundus images (mimic by CroppedUWF). In normal view field fundus images, the algorithm always focused on the blood vessels around the optic disc and macula. But in UWF fundus images, in addition to the region around the optic disc and macula, the algorithm also paid attention to the vessels away from the center. These regions are where we could not see in normal view field fundus images, which means UWF fundus images could provide more information to help the algorithms make a more precise prediction.

Noteworthy, the regions of interest were centered around retinal blood vessels, either in the posterior pole or the peripheral retina, which was consistent with the study by Wei et al. (2021), which developed a deep-learning-based anemia prediction model through retinal vessel OCT images. The relationship between anemia and retinal blood vessels could be explained for several reasons: 1) in severe anemia, retinopathy might develop because of the venous endothelial incompetence and decreased erythrocyte deformability secondary to hypoxia (Kirkham et al., 1971); 2) Choroidal thickness, which is an important indicator of ocular blood circulation, was significantly thinner in IDA patients than those of healthy subjects (Simsek et al., 2016). 3) IDA patients seemed to have significantly lower retinal capillary plexus density (Korkmaz et al., 2020).

As is known, anemia is a huge burden on health worldwide. Facing the situations of limited medical resources, ASModel_UWF may help clinicians to screen anemia with high efficiency and avoid unnecessary invasive blood sample collection. This ASModel_UWF might have promising prospects, which could be used to screen anemia in a large population or in rural areas. Given our previously accomplished function of deep-learning-based retinal break and retinal detachment recognition through UWF images (Zhang et al., 2021) and the renal function prediction by fundus images (Zhao et al., 2021), we might achieve the goal of screening and evaluating cardiovascular disease, renal function, nervous system diseases, and fundus diseases merely by a single shot of UWF images.

This study had several limitations. First, compared with the dataset from the United kingdom biobank, our sample size was relatively small, so we could not conduct subgroup analysis according to different types of anemia; second, this is a cross-sectional study, and the course of anemia and patients’ features could not be evaluated in detail; third, abnormal white blood cells, thrombocytopenia, and anemia were all the risk factors for retinopathy; we will evaluate the influence of WBC and blood platelet in future. Finally, UWF images are quite different from traditional fundus photographs (TFPs); we could translate the UWF images to the TFP domain, exploring if the performance would be improved.

## Conclusion

To our knowledge, this is the first study to show that a deep-learning model using UWF images could both predict hemoglobin concentration and screen anemia. The application of deep-learning to UWF images could facilitate a non-invasive and accurate screening for anemia in quite a large population and in rural areas. Further research studies are warranted to merge our previously developed deep-learning techniques in one module, to achieve the goal of screening and evaluating cardiovascular disease, renal function, nervous system diseases, and fundus diseases merely by a single shot of UWF images.

## Data Availability

The raw data supporting the conclusion of this article will be made available by the authors, without undue reservation.
