# Supplementary material for: Deep-Learning-Based Hemoglobin Concentration Prediction and Anemia Screening Using Ultra-Wide Field Fundus Images
Source: Front Cell Dev Biol. 2022 May 19;10:888268. doi: 10.3389/fcell.2022.888268 (PMC9160874; doi:10.3389/fcell.2022.888268)
Supplement: Supplementary file 1 [file Table1.DOCX]

| Suppl 1 Basic characteristics of the datasets | | | |
| --- | --- | --- | --- |
|  | Training dataset | Validation dataset | Test dataset |
| Total no. of images | 9221 | 577 | 1730 |
| No. of participants | 2445 | 213 | 565 |
| Age (years) | 44 (33-61) | 43 (26-61) | 43 (27-57) |
| Female (%) | 1381 (56.5%) | 119 (55.9%) | 313 (55.4%) |
| Hemoglobin (g·dl^-1^) | 13.6 (12.6-14.8) | 13.5 (12.5-14.9) | 13.5 (12.5-14.4) |
| Anaemia (0-12 g·dl^-1^ (F), 0-13 g·dl^-1^ (M)) | 1519 (16.5%) | 95(16.5%) | 215 (12.4%) |
| Age and hemoglobin concentration are presented as median values (interquartile range).  F=Female; M=Male. | | | |
